# Phase II trial of selective internal radiation therapy and systemic chemotherapy for liver-predominant metastases from pancreatic adenocarcinoma

**DOI:** 10.1186/s12885-015-1822-8

**Published:** 2015-10-26

**Authors:** Peter Gibbs, Cuong Do, Lara Lipton, David N. Cade, Michael J. Tapner, David Price, Geoff D. Bower, Richard Dowling, Meir Lichtenstein, Guy A. van Hazel

**Affiliations:** 1 Department of Medical Oncology, Royal Melbourne Hospital, Grattan Street, Parkville, VIC 3050 Australia; 2 Department of Medical Oncology, Western Hospital, Melbourne, Australia; 3 Sirtex Medical Limited, Sydney, Australia; 4 Perth Radiological Clinic, Mount Hospital, Perth, Australia; 5 Mount Nuclear Medicine, Mount Hospital, Perth, Australia; 6 Department of Radiology, Royal Melbourne Hospital, Melbourne, Australia; 7 Department of Nuclear Medicine, Royal Melbourne Hospital, Melbourne, Australia; 8 Perth Oncology, Mount Hospital, Perth, Australia

**Keywords:** Advanced, Liver, Metastases, Pancreas, Radioembolization, SIRT

## Abstract

**Background:**

This prospective, open-label phase II study assessed the impact of liver-directed therapy with selective internal radiation therapy (SIRT) and systemic chemotherapy on progression-free survival (PFS) in liver-dominant metastatic pancreatic adenocarcinoma.

**Methods:**

Patients received yttrium-90-labelled (^90^Y) resin microspheres (SIR-Spheres; Sirtex Medical Limited, Sydney, Australia) as a single procedure on day 2 of the first weekly cycle of 5-fluorouracil (5FU; 600 mg/m^2^) with the option to switch to gemcitabine (1000 mg/m^2^) after 8 weeks of 5FU. Statistical analysis was conducted using Microsoft Excel (Microsoft Corporation, Redmond, Washington, USA). The primary endpoint of the study was PFS in the liver, with a median of ≥16 weeks defined as the threshold for clinical significance. PFS and overall survival (OS) were summarised by the Kaplan-Meier method using non-parametric estimates of the survivor function.

**Results:**

Fourteen eligible patients were enrolled; ten had primary tumour in situ and eight had liver-only metastases. Patients received a median ^90^Y activity of 1.1 GBq and 8 weekly doses of 5FU; seven patients received a median of two doses of gemcitabine. Disease control in the liver was 93 % (two confirmed partial responses [PR], one unconfirmed PR, ten stable disease). Median reduction in cancer antigen 19–9 was 72 %. Median PFS was 5.2 months in the liver, which met the primary endpoint of the study, and 4.4 months at any site. PFS was prolonged in those with a resected primary compared with patients with primary in situ (median 7.8 vs. 3.4 months; *p* = 0.017). Median OS was 5.5 months overall and 13.6 months in patients with a resected primary. Grade 3/4 adverse events occurred in eight (57 %) patients during days 0–60. There was one sudden death and another patient who died from possible treatment-related liver failure 7.0 months after SIRT.

**Conclusions:**

SIRT and chemotherapy appears to be an effective treatment for liver metastases from pancreatic cancer, likely to be of most benefit in selected patients with a resected primary tumour and liver only disease. Significant toxicity was observed and the safety of this approach in patients with metastatic pancreatic cancer will need to be confirmed in subsequent studies. Further study is warranted with SIRT and modern chemotherapies.

**Trial registration:**

ACTRN12606000015549

**Electronic supplementary material:**

The online version of this article (doi:10.1186/s12885-015-1822-8) contains supplementary material, which is available to authorized users.

## Background

Pancreatic cancer is the fourth leading cause of cancer-related deaths in the USA and the fifth leading cause of cancer deaths in Europe with the incidence continuing to rise [1, 2]. The majority of patients have locally advanced and/or metastatic disease at presentation, resulting in a dismal 5-year survival rate of less than 5 % [3]. At the time that this study was conducted, gemcitabine was the most widely used systemic treatment for metastatic pancreatic cancer. In a phase III study, gemcitabine had a modest survival benefit of 5.6 months compared with 4.4 months for 5-fluorouracil (5FU) [4], and so the rationale for treatment with gemcitabine was primarily the alleviation of disease-related symptoms rather than extending overall survival (OS) [4]. Since then, the landmark European PRODIGE-4 trial with combined chemotherapy with folinic acid, fluorouracil, irinotecan and oxaliplatin (FOLFIRINOX) has extended the OS of patients with metastatic pancreatic cancer beyond 10 months (11.1 months vs. 6.8 months with gemcitabine) [5]. However, despite improvements in the control of locally advanced and metastatic disease with FOLFIRINOX [5–7], a recent published multicentre evaluation showed that Zapproximately one-third of patients were hospitalised due to adverse events [8] and it remains uncertain how widely this regimen will be used in the routine management of pancreatic cancer.

Liver metastases are a dominant cause of treatment failure in pancreatic cancer, occurring in 25–53 % of patients even after loco-regional control with chemo-radiation and surgical resection [9]. For patients with advanced disease, the PRODIGE-4 trial showed hepatic metastases and declining liver function (defined by albumin levels <3.5 g/dL) remain independent adverse prognostic factors for OS [5]. Consequently, liver-directed therapies, which improve disease control in the liver, may be of value in extending OS when combined with systemic chemotherapy.

Selective internal radiation therapy (SIRT) with yttrium-90 (^90^Y)-labelled microspheres is a loco-regional treatment, which has been evaluated in patients with hepatocellular carcinoma [10–12], as well as liver-dominant neuroendocrine tumours [13, 14], colorectal cancer (CRC) [15–18] and breast cancer [19–21]. To date, however, there have been very limited published data on SIRT in pancreatic cancer [22, 23]. Following the positive experience with SIRT in these primary and secondary hepatic neoplasms, we conducted a prospective study to assess whether SIRT, combined with 5FU, would extend PFS in the liver, and consequently OS, in patients with liver-only or liver-dominant advanced pancreatic cancer.

## Methods

### Study design

This was a prospective, open-label, multicentre, phase II trial to assess the safety and efficacy of SIRT combined with 5FU in patients with recently diagnosed liver-dominant metastases from pancreatic adenocarcinoma. Enrolled patients were to have liver metastases as the dominant clinical issue and the site of disease that threatened the patient’s life. This criterion was created with the expectation that enrolled patients would have bulky liver metastases, and small volume or no extra-hepatic disease.

Patients received SIRT using ^90^Y-resin microspheres (SIR-Spheres; Sirtex Medical Limited, Sydney, Australia) as a single procedure, 2 days after the first bolus injection of 5FU 600 mg/m^2^ (administered once weekly). Patients with on-going response could continue to receive chemotherapy for 16 weeks, until disease progression or unacceptable toxicity. At the time the protocol was written the standard treatment options for metastatic pancreatic cancer were gemcitabine and 5FU. Given the safety data for the combination of 5FU and SIRT and in order to avoid the well-documented radiosensitising effects of gemcitabine [24, 25] initial therapy was with 5FU alone, Investigators had the option after 8 weeks of 5FU to switch patients to gemcitabine 1000 mg/m^2^ (given weekly for 7 weeks followed by a 1-week rest, and thereafter weekly for 3 weeks, every 4 weeks) provided that the patient had not progressed on or experienced unacceptable toxicity from gemcitabine previously.

The principal aim of the study was to evaluate hepatic disease control, using PFS in the liver as the primary endpoint. The combination of SIRT and chemotherapy would be considered to be of clinical significance if the median PFS in the liver was ≥16 weeks. This was based on data from a study of 5FU alone, where more than 70 % of patients had disease progression by 2 months [4], suggesting that if median PFS in the liver was more than twice this it would suggest significant impact from the addition of SIRT. The secondary endpoints were: safety and toxicity, PFS at any site, best objective response rate in the liver and at any site, site of disease progression and OS.

The study conformed to the World Medical Association Declaration of Helsinki and the Australian National Health and Medical Research Council statement on human experimentation. Prior approval of the study protocol was received from each institute’s Human Research Ethics Committee (Melbourne Health Ethics Committee study 2006.124 and Mount Hospital Ethics Committee study EC35.3).

### Patients

Patients were enrolled at two centres (Mount Medical Centre, Perth, Australia; Western Hospital, Melbourne, Australia) between October 2006 and November 2009. All patients were fully informed of the nature of the trial and signed an informed consent document.

Patients were included in the study if they were 18 years of age or older, with a life expectancy of ≥2 months without any active treatment, had a World Health Organization (WHO) performance status of 0 or 1, and a diagnosis of pancreatic adenocarcinoma. At the time of inclusion, the liver had to be the dominant site of disease, impacting on patients’ health-related quality of life and/or survival; low-volume extra-hepatic metastases and/or an intact primary cancer were permitted. Previous chemotherapy, either as adjuvant treatment or first-line therapy for metastatic disease was permitted. All laboratory parameters had to be within the defined limits for the safe delivery of SIRT, i.e. neutrophil count >1.5 × 10^9^/L; platelets >100 × 10^9^/L; creatinine <150 μmol/L; bilirubin ≤1 × upper limit of normal (ULN); albumin ≥3 g/dL. Female patients were either postmenopausal, sterile, or if sexually active using an acceptable method of contraception. Male patients, if sexually active (and not surgically sterile) and having a pre-menopausal partner, were required to use an acceptable method of contraception.

Patients were excluded with evidence of ascites, cirrhosis or portal hypertension (as determined by clinical or radiological assessment), occlusion of the main portal vein, central nervous system metastases, prior radiotherapy that included the liver in the treatment field, prior treatment with an investigational agent within 30 days of SIRT, or evidence of any concurrent condition that, in the opinion of the investigator, would render the patient ineligible for treatment according to this protocol.

### Assessment and data handling

All baseline assessments were carried out within 29 days of enrolment, including serum cancer antigen 19-9 (CA19-9), and computed tomography (CT) or magnetic resonance imaging (MRI) to assess the extent of disease in the abdomen and chest. The percentage tumour burden within the liver was determined using the baseline CT/MRI scan, utilising validated tumour volumetry software (MeVis Distant Services, Bremen, Germany). Patients underwent a baseline hepatic angiogram to map the vascular anatomy of the liver. Technetium-99 m macroaggregated albumin was used as a surrogate for ^90^Y-resin microspheres during the pre-treatment planning to determine the presence and magnitude of arterio-venous shunting to the lungs so that the lung radiation exposure could be kept within safe limits (<25 Gy) and the activity of ^90^Y administered was adjusted accordingly [25]. The calculated activity of ^90^Y to be implanted was determined from tables provided by the manufacturer, based on a modification of the Body Surface Area formula and adjusted for the extent of lung shunting for each patient (see Additional file 1).

Post-SIRT, patients were evaluated every 4 weeks and tumour response was assessed every 8 weeks until disease progression in the liver according to Response Evaluation Criteria In Solid Tumours (RECIST) version 1.0. Complete response or partial response (PR) of liver metastases were confirmed by a further CT scan performed after 4 weeks. Patients were analysed according to the presence or absence of the primary tumour in situ and the presence or absence of extra-hepatic metastases.

PFS in the liver was defined as the interval between trial entry and the date of tumour progression (based on RECIST) or death, whichever occurred sooner. OS was defined as the interval between enrolment and the date of death.

Adverse events were recorded from consent until 30 days after the last dose of protocol chemotherapy was administered. At the time of their occurrence, the causal relationship between the adverse events and the protocol therapy was recorded by the investigator. All adverse events were graded using the National Cancer Institute Common Terminology Criteria for Adverse Events (NCI-CTCAE) version 3.0. Adverse events are presented according to time of occurrence: from enrolment up to day 60 or beyond day 60.

Statistical analysis was conducted using Microsoft Excel (Microsoft Corporation, Redmond, Washington, USA). PFS and OS was summarised by the Kaplan-Meier method using nonparametric estimates of the survivor function.

## Results

### Baseline characteristics and treatment

Fifteen patients were enrolled and received protocol therapy. One patient was excluded from the analysis after the histopathology report confirmed retrospectively that the liver lesion was derived from a pancreatic neuroendocrine tumour. Patient and disease characteristics for the remaining 14 patients are detailed in Table 1. Patients entered the study a median of 13 days after diagnosis of metastatic pancreatic cancer. Most patients had a good performance status, either WHO 0 (71 %) or 1 (29 %). The primary cancer was in situ in ten patients (71 %); six of these patients had liver-only metastases, and four had extrahepatic disease/metastases (EHD). The primary tumour had been resected in four patients (two with liver-only disease; two with EHD). Three patients had received prior chemotherapy (three received gemcitabine, two 5FU, one carboplatin); all three had liver-only metastases, two with a resected primary. The tumour burden in the liver varied between 1 and 37 % of the liver volume (mean 14 %), with bilobar involvement in all patients.

**Table 1:** Baseline patient and disease characteristics

Characteristics		Patients	
		Number	Percentage
Gender	Male	6	43 %
	Female	8	57 %
Age, years; median (range)		62 (48–76)	
WHO performance status	0	10	71 %
	1	4	29 %
Time from diagnosis of metastatic pancreas cancer to trial entry, ^a^days; median (range)		13 (5–434)	
Cancer stage at diagnosis	TX	4	29 %
	T2	6	43 %
	T3	2	14 %
	T4	2	14 %
Primary tumour in situ	Yes	10	71 %
	No	4	29 %
Metastases	Liver only	8	57 %
	Liver and lung	2	14.5 %
	Liver and lymph nodes	2	14.5 %
	Liver, lung and lymph nodes	1	7 %
	Liver, lung, lymph nodes, soft tissue	1	7 %
Number of metastatic sites; median (range)		3 (1–5)	
CA19-9, ^b^U/mL; median (range)		3480 (33–280,000)	
>ULN		13	93 %
Prior lines of chemotherapy for metastatic disease	0	13	93 %
	1	1	7 %

^a^Trial entry defined as day of informed consent

^b^*N* = 13 patients with elevated CA19-9 baseline levels (ULN 37 U/mL)

All patients received SIRT 2 days after the first weekly dose of 5FU. The median prescribed and administered activity of ^90^Y-resin microspheres was 1.1 GBq (range 0.7–2.0 GBq). ^90^Y-resin microspheres were administered by selective injection to left and right hepatic arteries except in one patient who did not receive SIRT in the right lobe due to an unfavourable hepatic arterial anatomy that precluded catheter access. Median follow-up from study entry was 5.9 months (range 1.4–16.2 months). Patients received a median of 8 weekly doses of 5FU (median 600 mg/m^2^; range 350–638 mg/m^2^). Seven patients (50 %), one of whom had progressive disease, received a median of 2 doses (range 2–10) of gemcitabine (median 1000 mg/m^2^; range, 600–1000 mg/m^2^), an average of 3.7 months (range 2.1–8.1 months) after the start of 5FU.

### Adverse events

Adverse events are listed in Table 2A and B. The majority of adverse events occurred during the first 60 days of therapy.

**Table 2:** Adverse events. Adverse events (by NCI-CTCAE v.3 grade) recorded up to 60 days after the start of protocol therapy, from 61 days onwards and across the whole study period (*n* = 14)

Category/event	Day 1 to 60 (*n* = 14)		Day 61 onwards (*n* = 12)		Day 1 to last assessment (*n* = 14)	
	Grade 1–2	Grade ≥3	Grade 1–2	Grade ≥3	Grade 1–2	Grade ≥3
A.						
Gastrointestinal						
Nausea	10	0	4	1	10	1
Vomiting	7	0	1	0	8	0
Anorexia	5	0	5	2	6	2
Diarrhoea	4	0	1	1	4	1
Stomatitis	1	0	2	0	2	0
Mucositis	2	0	0	0	2	0
Constipation	2	0	1	0	2	0
Pain						
Abdominal pain	2	0	1	0	3	0
Pain (non-abdominal)	0	1	0	0	0	1
Constitutional Symptoms						
Fatigue	6	2	5	5	4	6
Fever	3	0	0	0	3	0
Hepatobiliary/Pancreas						
Ascites	0	1	0	3	0	3
Jaundice	0	0	0	1	0	1
Liver failure	0	0	0	1	0	1
Neurology						
Neuropathy	3	0	1	0	3	0
Pulmonary/Upper Respiratory						
Dyspnoea	1	1	1	1	1	1
Bruising	1	0	2	0	2	0
Pneumonia	0	1	1	0	0	1
Vascular						
Pulmonary embolism	0	1	0	0	0	1
Deep vein thrombosis	0	1	0	1	0	1
Dermatology/Skin						
Dry skin/cracked skin	1	1	0	1	1	1
Haemorrhage/Bleeding						
Epistaxis	1	0	2	0	2	0
Ocular/Visual						
Epiphora	3	0	3	0	3	0
B.						
Biochemical/Laboratory						
Hyperbilirubinemia	5	3	2	6	4	7
Albumin	9	1	8	1	11	1
Alkaline phosphatase	4	1	8	0	8	1
Alanine transaminase	8	0	5	0	10	0
Aspartate aminotransferase	3	0	5	0	6	0
Blood/Bone Marrow						
Haemoglobin	7	0	3	0	8	0
Platelets	4	2	5	2	4	4
Leukocytes	8	1	2	0	8	1
Neutrophils	2	2	1	1	2	2

A) Any grade 1–2 treatment-related adverse clinical events occurring in >10 % of patients and all grade 3–4 treatment-related adverse clinical events. B) All-cause laboratory events

Early events (days 0–60): grade 3/4 adverse clinical and/or laboratory events occurred in 8 (57 %) patients during this period. Nine patients (64 %) had no grade 3 or higher treatment-related clinical adverse events, and there was no grade 3 or 4 abdominal pain, nausea, vomiting or diarrhoea during this period. Two patients (14 %) developed grade 3 fatigue which may have been treatment-related. Grade 3 biochemical toxicities were observed in two patients (14 %) and significant haematological events in four patients (28 %): grade 3 neutropenia (two patients; 14 %) and grade 3 thrombocytopenia (two patients; 14 %).

Later events (after 60 days): six patients (50 %) had grade 3 or higher treatment-related clinical adverse events, four of whom had switched to gemcitabine, with biochemical toxicities reported in six patients (50 %, three on gemcitabine) and hematologic events in three patients (25 %, all on gemcitabine).

Death without documented progression occurred in two patients. The first patient died suddenly 1.5 months after study entry and within 28 days of SIRT; the patient had no adverse events greater than grade 1 severity, laboratory tests were within normal limits but CA19-9 had increased by 293 % from baseline. At enrolment, this patient had a T4 primary tumour in situ with metastases to the liver, lungs and lymph nodes. The second patient, who died 7.0 months after study entry, presented with liver-only metastases following prior resection of a T2 primary cancer. The patient died from hepatic failure, considered to possibly be due to radioembolization-induced liver disease (REILD), although the role of prior gemcitabine and herbal remedies prior to protocol therapy should also be considered. The patient had also received external beam radiotherapy (45 Gy in 25 fractions) to the pancreas.

### Treatment response and survival

**Fig. 1:**
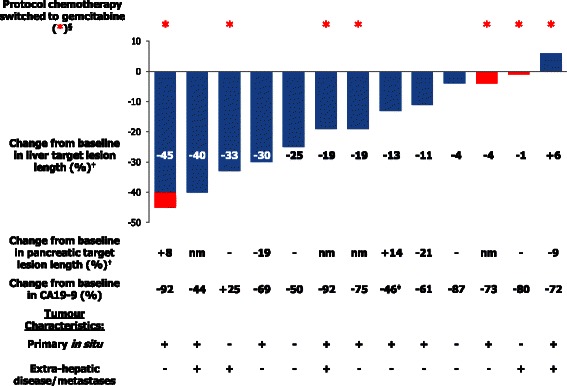
Percentage change from baseline in the sum of index lesions in the liver. Waterfall plot of percentage change from baseline in the sum of index lesions in the liver, with pancreatic lesion response, CA19-9 response and tumour characteristics. ^§^ Patients switching protocol chemotherapy to gemcitabine 2.1–8.1 months after the start of 5FU (red asterisk); ^†^ Tumour response by RECIST v1.0 (change while on 5FU in blue; change while on gemcitabine in red); nm: non-measurable disease; ^‡^ baseline value < ULN (excluded from analysis of mean change in CA19-9)

**Fig. 2:**
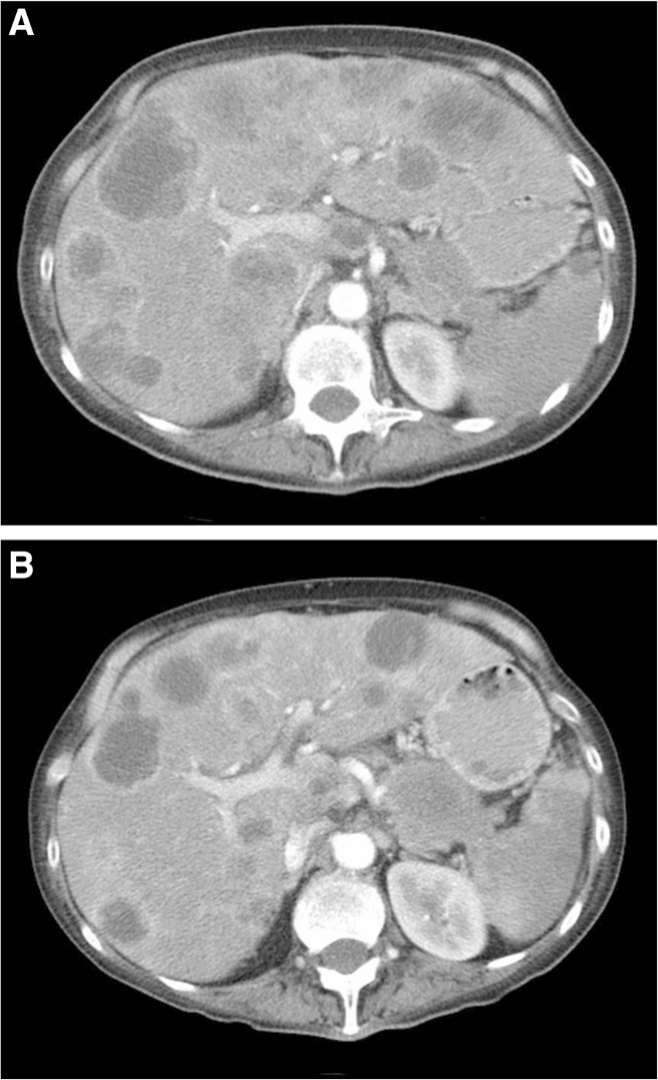
Tumour response in a patient with liver-only metastases from primary pancreatic adenocarcinoma. **a** Contrast-enhanced CT scan prior to SIRT + 5FU. **b**) Follow-up contrast-enhanced CT scan 3 months post-SIRT + 5FU, and prior to gemcitabine, demonstrates a partial response (40 % reduction in hepatic tumour burden), as assessed according to RECIST v1.0

Individual best response within the liver (according to RECIST) is presented in the waterfall plot together with the tumour characteristics and CA19-9 response (Fig. 1). A PR in the liver was recorded in three patients (21 %) (2 confirmed; 1 unconfirmed) and stable disease (SD) in ten patients (71 %), giving a liver disease control rate of 93 %. CT scans of a patient with a partial response are shown in Fig. 2. Best response (at all sites) was 1 PR (7 %), SD in nine patients (64 %) and progression in four patients (29 %). Median reduction in CA19-9 was 72 %. Switching to gemcitabine did not appear to contribute to the initial response.

**Fig. 3:**
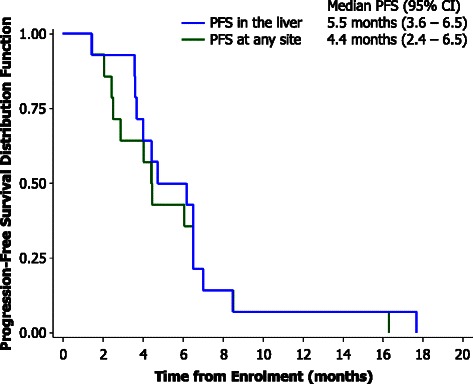
Kaplan-Meier analysis of PFS and OS. 3) PFS in the liver and at any site. 4) PFS at any site stratified by the presence or absence of the primary tumour in situ. 5) OS. 6) OS stratified by the presence or absence of the primary tumour in situ. 7) OS stratified by the presence of liver-only metastases or liver plus EHD

The median liver PFS was 5.2 months (range 1.4–17.7 months), which exceeded the pre-specified threshold for clinical significance, and 4.4 months (range 1.4–16.3 months) at any site (Fig. 3). The median time to progression in the liver was derived by per protocol follow-up scans on all patients until progression of liver metastases, including patients who had progressed outside the liver as the first site of progression and may have commenced a second line of chemotherapy. PFS at any site was shorter in patients with advanced primary tumour in situ compared with those who had their primary tumour resected (median 3.4 vs. 7.8 months; *p* = 0.017; Fig. 4). Two patients with liver-only metastases and a resected primary had an overall PFS of 16.3 and 7.0 months, respectively.

The first site of progression is listed in Table 3. The liver was the first site of progression in two patients: one with new lesions at 6.5 months and one with progression of existing liver lesions at 8.5 months after enrolment. Three patients (21 %) deteriorated clinically without documented evidence of disease progression. Two patients (13 %) were withdrawn from study treatment due to adverse events: one with grade 2 nausea/vomiting 3.6 months after study entry and 1 week after commencing gemcitabine, and the other with obstructive jaundice and ascites, at 2.7 months post-enrolment and 2 weeks after switching to gemcitabine.

**Table 3:** Site of first progression (*n* = 14)

Location of first progression	Patients	
	Number	Percentage
Documented progression on CT		
Liver	2	20
Lung	2	13
Lymph node	1	7
Peritoneum	1	7
Pleural effusion	1	7
No progression documented		
Clinical deterioration	3	20
Death without progression	2	13
Withdrawn due to adverse events	2	13

Median OS was 5.5 months (range 1.4–19.5 months) for the entire cohort (Fig. 5) and 12.2 months (range 7.0–17.7 months) for patients with disease confined to the liver. Unplanned subgroup analysis revealed significantly longer survival in patients whose primary tumour had been resected (*n* = 4; median 13.6 vs. 4.2 months; *p* = 0.015; Fig. 6). For patients with liver-only metastases (*n* = 8), median survival was 6.6 months (range 3.6–17.7 months; Fig. 7). Where patients had EHD (*n* = 6), the median survival was 4.6 months (range 1.4–19.5 months). A summary of the characteristics of patients alive after 12 months is shown in Table 4.

**Table 4:** Characteristics of patients who survived greater than 12 months

Patient	Stage at diagnosis^a^	Status of primary tumour	Site of metastases	PFS in the liver (months)	PFS (months)	Survival (months)	Site of first disease progression
Patient 1	Tx	Primary in situ	Liver only	6.5	6.5	16.2	Liver
Patient 2	T2	Prior resection	Liver only	17.7	16.3	17.7	Pleural effusion
Patient 3	T3	Prior resection	Liver and lymph nodes	8.5	8.5	19.5	Liver

^a^According to United Network for Organ Sharing staging criteria

## Discussion

Metastatic pancreatic cancer carries a very poor prognosis. Progress has been frustratingly slow with numerous agents in combination with gemcitabine demonstrating promise in phase II studies, but minimal impact on OS in subsequent phase III randomised trials. Limited benefit has been demonstrated with the addition of oxaliplatin [26], cisplatin [27], capecitabine [28, 29], cetuximab [30], bevacizumab [31] or erlotinib [32] to gemcitabine. A recent study demonstrated a significant improvement in OS with FOLFIRINOX, but uncertainty remains as to whether this can be safely achieved in routine clinical practice [5].

In this first prospective study of the safety and efficacy of SIRT in advanced pancreatic cancer, 5FU rather than gemcitabine was administered concomitantly with ^90^Y-resin microspheres, thereby circumventing any potential adverse events associated with gemcitabine radiosensitisation of non-target tissue. Overall, the spectrum of adverse events in this study associated with SIRT (characterised by mild-to-moderate abdominal pain, nausea, and transient changes in liver function) and 5FU (neutropenia and thrombocytopenia) were similar to those reported in several previous trials in patients with liver metastases from CRC, in which this combination has been demonstrated to be safe [15, 16]. The incidence of grade 3/4 haematological toxicities (14 %) was consistent with the past experience with SIRT and 5FU [15, 16]. Patients who received gemcitabine no sooner than 8 weeks post-SIRT experienced a similar rate of adverse events as would be expected from gemcitabine therapy alone [4], without any evidence of REILD [33]; suggesting that gemcitabine can safely be given after SIRT.

In this study, one responding patient on 5FU developed signs suggestive of REILD and died at 7.0 months after treatment. REILD is defined as jaundice and ascites in the absence of tumour progression or bile duct obstruction commencing within 8 weeks of SIRT [33], so this event occurred well outside that window. Both rising bilirubin levels (from grade 2 on day 39 to grade 4 on day 102) and elevated alkaline phosphatase levels (which were greater than grade 1 from day 116 onwards) are recognised hallmarks of REILD [33]; although the clinical picture in this patient was complicated by use of herbal remedies [34] and gemcitabine pre-SIRT, which may have contributed to liver failure in this case as may have disease progression. The sudden death observed in one other patient was considered unlikely to be related to SIRT as sudden deaths on SIRT have not previously been reported, and the only likely cause of treatment related death (liver failure) is usually of slow onset with clinical and laboratory signs evident well in advance of patients dying.

Tumour response within the liver was encouraging, with 21 % of patients (three out of 14) achieving a confirmed or unconfirmed PR and 71 % (ten out of 14) achieving SD, by RECIST criteria, for a disease control rate of 93 %. As shown in Fig. 1, the size of the liver lesions diminished in all but one of the 13 patients who had post-SIRT imaging at 8 weeks intervals and no patient had progressive disease within the liver on initial follow-up CT imaging. With limited radiological response on gemcitabine, it would seem reasonable to conclude that the recorded response was largely due to the protocol treatment rather than subsequent gemcitabine.

The imaging results are also corroborated by the decline in CA19-9 observed in 12 of 13 patients with an elevated CA19-9 at baseline, including all but one of those with EHD. Similar to the experience in SIRT-treated CRC liver metastases [35, 36], the decline in tumour marker was rapid and appeared to predict later CT response and PFS.

The results of our phase II study compare favourably to a time to progression in the randomised controlled trial by Burris et al. of 0.9 months for 5FU alone, and 2.3 months for gemcitabine and response rate with 5FU alone of 0 and 19 % SD. Subsequently Cunningham et al. has recorded small incremental improvements in PFS with gemcitabine combined with capecitabine compared with gemcitabine alone (5.3 vs. 3.8 months), which have been accompanied by a small survival benefit (6.2 vs. 7.1 months) [29]. The median PFS reported for patients treated with FOLFIRINOX in the recent randomised study was 6.4 months [5]. Significantly however, the studies by Cunningham et al. [28] and that of Burris et al. [4] included many patients with locally advanced disease (29 and 26 % of patients, respectively), for whom the median PFS would be expected to be superior, whereas the current study and the FOLFIRINOX study only included patients with metastatic disease.

As expected for a liver-directed therapy, studies of SIRT in patients with CRC have demonstrated better outcomes in patients with disease confined to the liver [18, 37]. In the current trial, outcomes likewise appeared related to extent of disease outside the liver, with the best results seen in the two patients with liver-only disease (OS of 7.0 and 17.7 months) and the worst outcomes in the four patients with an intact primary and liver metastases plus EHD (median OS of 4.2 months). These results suggest that further studies of SIRT in pancreatic cancer liver metastases should be confined to the population of patients with liver-only disease who have had their primary lesion resected or who have well-controlled primary disease. These analyses should not be used to select patients for treatment outside of clinical trials, as SIRT remains an experimental treatment option in this disease type.

## Conclusions

The data obtained from this study of the combination of SIRT and 5FU in the treatment of liver metastases from primary pancreatic cancer demonstrated evidence of effective disease control of liver metastases from pancreatic adenocarcinoma, with a disease control rate of 93 % and a liver PFS of 5.2 months. However, the combination of SIRT and 5FU resulted in a toxicity profile that was significant and the safety of this approach in patients with metastatic pancreatic cancer will need to be confirmed in subsequent studies. This combination of therapy is likely to be of most benefit in selected patients with a resected primary tumour and liver only disease. Ultimately though, randomised trials will be needed to prove the role of SIRT in combination with chemotherapy in metastatic pancreatic cancer, and to define the patients who will most benefit from this treatment. Strategies combining SIRT with gemcitabine are likely to be limited by the doses of gemcitabine that could be given safely with SIRT, without compromising its systemic activity. Several studies in CRC have demonstrated that SIRT can be safely combined with FOLFOX [15–17] and with irinotecan [38], suggesting that initial cycles with FOLFOX or irinotecan might be an attractive strategy.

## Human research and ethics committees approval

This study was approved by the ethics committees of Melbourne Health, Parkville, Australia (for patients enrolled at Western Hospital) and Mount Hospital, Perth, Australia (for patients enrolled at Mount Hospital)

## Additional file

Additional file 1: **Dosimetry tables for** ^**90**^**Y-resin microspheres.** The following tables (**Table S1A to S1C**) provided by Sirtex Medical Limited, Sydney, Australia were used in this phase II study to guide the dosimetry for ^90^Y-resin microspheres (SIR-Spheres). The tables, based a modification of the Body Surface Area (BSA) formula, calculated the activity in gigabecquerels (GBq) of ^90^Y which was to be implanted determined from the BSA and percentage tumour involvement of whole liver for each patient. Three tables were provided so that the implanted activity of ^90^Y could be further adjusted for each patient according to extent of lung shunting (0–10 %; 11–15 %; 16–20 %). The tables provide a more user-friendly method for calculating the dosimetry for ^90^Y-resin microspheres, while at the same including slight modifications to the dosing, in order to improve the safety of this procedure in patients with either a very low (<10 %) or high (>60 %) tumour volume in the liver. (DOCX 43 kb)

## Abbreviations

5FU: 5-fluorouracil
Y: Yttrium-90
CA19-9: Cancer antigen 19–9
CRC: Colorectal cancer
CT: Computed tomography
EHD: Extrahepatic disease/metastases
FOLFIRINOX: Folinic acid, fluorouracil, irinotecan, oxaliplatin
FOLFOX: Folinic acid, fluorouracil, oxaliplatin
mCRC: Metastatic colorectal cancer
MRI: Magnetic resonance imaging
NCI-CTCAE: National Cancer Institute Common Terminology Criteria for Adverse Events
OS: Overall survival
PFS: Progression-free survival
PR: Partial response
RECIST: Response Evaluation Criteria In Solid Tumours
REILD: Radioembolization-induced liver disease
SD: Stable disease
SIRT: Selective internal radiation therapy
ULN: Upper limit of normal
WHO: World Health Organization
