# Supplementary material for: Phase II trial of selective internal radiation therapy and systemic chemotherapy for liver-predominant metastases from pancreatic adenocarcinoma
Source: BMC Cancer. 2015 Oct 26;15:802. doi: 10.1186/s12885-015-1822-8 (PMC4624193; doi:10.1186/s12885-015-1822-8)
Supplement: Additional file 1: — Dosimetry tables for 90Y-resin microspheres. The following tables (Table S1A to S1C) provided by Sirtex Medical Limited, Sydney, Australia were used in this phase II study to guide the dosimetry for 90Y-resin microspheres (SIR-Spheres). The tables, based a modification of the Body Surface Area (BSA) formula, calculated the activity in gigabecquerels (GBq) of 90Y which was to be implanted determined from the BSA and percentage tumour involvement of whole liver for each patient. Three tables were provided so that the implanted activity of 90Y could be further adjusted for each patient according to extent of lung shunting (0–10 %; 11–15 %; 16–20 %). The tables provide a more user-friendly method for calculating the dosimetry for 90Y-resin microspheres, while at the same including slight modifications to the dosing, in order to improve the safety of this procedure in patients with either a very low (<10 %) or high (>60 %) tumour volume in the liver. (DOCX 43 kb) [file 12885_2015_1822_MOESM1_ESM.docx]

**Additional file 1**

This additional file formed part of the original submission.

Supplement to:

Phase II trial of selective internal radiation therapy and systemic chemotherapy for liver-predominant metastases from pancreatic adenocarcinoma.

P Gibbs, C Do, L Lipton, DN Cade, MJ Tapner, D Price, GD Bower, R Dowling, M Lichtenstein and GA van Hazel.

**Dosimetry tables for ^90^Y-resin microspheres.** The following tables (**Table S1A to S1C**) provided by Sirtex Medical Limited, Sydney, Australia were used in this phase II study to guide the dosimetry for ^90^Y-resin microspheres (SIR-Spheres). The tables, based a modification of the Body Surface Area (BSA) formula, calculated the activity in gigabecquerels (GBq) of ^90^Y which was to be implanted determined from the BSA and percentage tumour involvement of whole liver for each patient. Three tables were provided so that the implanted activity of ^90^Y could be further adjusted for each patient according to extent of lung shunting (0–10%; 11–15%; 16–20%). The tables provide a more user-friendly method for calculating the dosimetry for ^90^Y-resin microspheres, while at the same including slight modifications to the dosing, in order to improve the safety of this procedure in patients with either a very low (<10%) or high (>60%) tumour volume in the liver.

**Table S1A. Activity calculator** **for patients with 0–10% liver-to-lung shunt ratio.** Activity values are given in GBq. BSA = body surface area.

| **BSA** | **Percentage tumour involvement (%)** | | | | | | | | | | | | | | | |
| --- | --- | --- | --- | --- | --- | --- | --- | --- | --- | --- | --- | --- | --- | --- | --- | --- |
|  | **0**–**5** | **6**–**10** | **11**–**15** | **16**–**20** | **21**–**25** | **26**–**30** | **31**–**35** | **36**–**40** | **41**–**45** | **46**–**50** | **51**–**55** | **56**–**60** | **61**–**65** | **66**–**70** | **71**–**75** | **76**–**80** |
| **1.30–1.35** | 0.7 | 0.8 | 1.0 | 1.2 | 1.3 | 1.4 | 1.5 | 1.5 | 1.5 | 1.5 | 1.4 | 1.3 | 1.3 | 1.2 | 1.1 | 1.1 |
| **1.36–1.40** | 0.7 | 0.9 | 1.0 | 1.2 | 1.3 | 1.4 | 1.5 | 1.5 | 1.5 | 1.5 | 1.5 | 1.4 | 1.3 | 1.2 | 1.2 | 1.1 |
| **1.41–1.45** | 0.7 | 0.9 | 1.1 | 1.2 | 1.4 | 1.5 | 1.6 | 1.6 | 1.6 | 1.6 | 1.5 | 1.4 | 1.4 | 1.3 | 1.2 | 1.2 |
| **1.46–1.50** | 0.8 | 0.9 | 1.1 | 1.3 | 1.4 | 1.5 | 1.6 | 1.7 | 1.7 | 1.6 | 1.6 | 1.5 | 1.4 | 1.3 | 1.3 | 1.2 |
| **1.51–1.55** | 0.8 | 1.0 | 1.1 | 1.3 | 1.5 | 1.6 | 1.7 | 1.7 | 1.7 | 1.7 | 1.6 | 1.5 | 1.5 | 1.4 | 1.3 | 1.2 |
| **1.56–1.60** | 0.8 | 1.0 | 1.2 | 1.4 | 1.5 | 1.6 | 1.7 | 1.8 | 1.8 | 1.7 | 1.7 | 1.6 | 1.5 | 1.4 | 1.3 | 1.3 |
| **1.61–1.65** | 0.8 | 1.0 | 1.2 | 1.4 | 1.6 | 1.7 | 1.8 | 1.8 | 1.8 | 1.8 | 1.7 | 1.6 | 1.6 | 1.5 | 1.4 | 1.3 |
| **1.66–1.70** | 0.9 | 1.1 | 1.3 | 1.4 | 1.6 | 1.7 | 1.8 | 1.9 | 1.9 | 1.8 | 1.8 | 1.7 | 1.6 | 1.5 | 1.4 | 1.4 |
| **1.71–1.75** | 0.9 | 1.1 | 1.3 | 1.5 | 1.7 | 1.8 | 1.9 | 1.9 | 1.9 | 1.9 | 1.8 | 1.7 | 1.6 | 1.5 | 1.5 | 1.4 |
| **1.76–1.80** | 0.9 | 1.1 | 1.3 | 1.5 | 1.7 | 1.8 | 1.9 | 2.0 | 2.0 | 1.9 | 1.9 | 1.8 | 1.7 | 1.6 | 1.5 | 1.4 |
| **1.81–1.85** | 0.9 | 1.1 | 1.4 | 1.6 | 1.8 | 1.9 | 2.0 | 2.0 | 2.0 | 2.0 | 1.9 | 1.8 | 1.7 | 1.6 | 1.5 | 1.5 |
| **1.86–1.90** | 1.0 | 1.2 | 1.4 | 1.6 | 1.8 | 1.9 | 2.0 | 2.1 | 2.1 | 2.1 | 2.0 | 1.9 | 1.8 | 1.7 | 1.6 | 1.5 |
| **1.91–1.95** | 1.0 | 1.2 | 1.4 | 1.7 | 1.9 | 2.0 | 2.1 | 2.1 | 2.1 | 2.1 | 2.0 | 1.9 | 1.8 | 1.7 | 1.6 | 1.6 |
| **1.96–2.00** | 1.0 | 1.2 | 1.5 | 1.7 | 1.9 | 2.1 | 2.2 | 2.2 | 2.2 | 2.2 | 2.1 | 2.0 | 1.9 | 1.8 | 1.7 | 1.6 |
| **2.01–2.05** | 1.0 | 1.3 | 1.5 | 1.7 | 1.9 | 2.1 | 2.2 | 2.3 | 2.3 | 2.2 | 2.1 | 2.0 | 1.9 | 1.8 | 1.7 | 1.6 |
| **2.06–2.10** | 1.1 | 1.3 | 1.6 | 1.8 | 2.0 | 2.2 | 2.3 | 2.3 | 2.3 | 2.3 | 2.2 | 2.1 | 2.0 | 1.9 | 1.8 | 1.7 |
| **2.11–2.15** | 1.1 | 1.3 | 1.6 | 1.8 | 2.0 | 2.2 | 2.3 | 2.4 | 2.4 | 2.3 | 2.2 | 2.1 | 2.0 | 1.9 | 1.8 | 1.7 |
| **2.16–2.20** | 1.1 | 1.4 | 1.6 | 1.9 | 2.1 | 2.3 | 2.4 | 2.4 | 2.4 | 2.4 | 2.3 | 2.2 | 2.1 | 1.9 | 1.8 | 1.8 |
| **2.21–2.25** | 1.1 | 1.4 | 1.7 | 1.9 | 2.1 | 2.3 | 2.4 | 2.5 | 2.5 | 2.4 | 2.3 | 2.2 | 2.1 | 2.0 | 1.9 | 1.8 |
| **2.26–2.30** | 1.2 | 1.4 | 1.7 | 2.0 | 2.2 | 2.4 | 2.5 | 2.5 | 2.5 | 2.5 | 2.4 | 2.3 | 2.2 | 2.0 | 1.9 | 1.8 |
| **2.31–2.35** | 1.2 | 1.5 | 1.7 | 2.0 | 2.2 | 2.4 | 2.5 | 2.6 | 2.6 | 2.5 | 2.5 | 2.3 | 2.2 | 2.1 | 2.0 | 1.9 |
| **2.36–2.40** | 1.2 | 1.5 | 1.8 | 2.0 | 2.3 | 2.5 | 2.6 | 2.6 | 2.6 | 2.6 | 2.5 | 2.4 | 2.3 | 2.1 | 2.0 | 1.9 |
| **2.41–2.45** | 1.2 | 1.5 | 1.8 | 2.1 | 2.3 | 2.5 | 2.6 | 2.7 | 2.7 | 2.6 | 2.6 | 2.4 | 2.3 | 2.2 | 2.0 | 1.9 |
| **2.46–2.50** | 1.3 | 1.5 | 1.8 | 2.1 | 2.4 | 2.6 | 2.7 | 2.8 | 2.8 | 2.7 | 2.6 | 2.5 | 2.4 | 2.2 | 2.1 | 2.0 |

**Table S1B. Activity calculator** **for patients with 11–15% liver-to-lung shunt ratio.** Activity values are given in GBq. BSA = body surface area.

| **BSA** | **Percentage tumour involvement (%)** | | | | | | | | | | | | | | | |
| --- | --- | --- | --- | --- | --- | --- | --- | --- | --- | --- | --- | --- | --- | --- | --- | --- |
|  | **0**–**5** | **6**–**10** | **11**–**15** | **16**–**20** | **21**–**25** | **26**–**30** | **31**–**35** | **36**–**40** | **41**–**45** | **46**–**50** | **51**–**55** | **56**–**60** | **61**–**65** | **66**–**70** | **71**–**75** | **76**–**80** |
| **1.30–1.35** | 0.7 | 0.8 | 1.0 | 1.2 | 1.3 | 1.4 | 1.5 | 1.5 | 1.5 | 1.5 | 1.4 | 1.3 | 1.3 | 1.2 | 1.1 | 1.1 |
| **1.36–1.40** | 0.7 | 0.9 | 1.0 | 1.2 | 1.3 | 1.4 | 1.5 | 1.5 | 1.5 | 1.5 | 1.5 | 1.4 | 1.3 | 1.2 | 1.2 | 1.1 |
| **1.41–1.45** | 0.7 | 0.9 | 1.1 | 1.2 | 1.4 | 1.5 | 1.6 | 1.6 | 1.6 | 1.6 | 1.5 | 1.4 | 1.4 | 1.3 | 1.2 | 1.2 |
| **1.46–1.50** | 0.8 | 0.9 | 1.1 | 1.3 | 1.4 | 1.5 | 1.6 | 1.7 | 1.7 | 1.6 | 1.6 | 1.5 | 1.4 | 1.3 | 1.3 | 1.2 |
| **1.51–1.55** | 0.8 | 1.0 | 1.1 | 1.3 | 1.5 | 1.6 | 1.7 | 1.7 | 1.7 | 1.7 | 1.6 | 1.5 | 1.5 | 1.4 | 1.3 | 1.2 |
| **1.56–1.60** | 0.8 | 1.0 | 1.2 | 1.4 | 1.5 | 1.6 | 1.7 | 1.8 | 1.8 | 1.7 | 1.7 | 1.6 | 1.5 | 1.4 | 1.3 | 1.3 |
| **1.61–1.65** | 0.8 | 1.0 | 1.2 | 1.4 | 1.6 | 1.7 | 1.8 | 1.8 | 1.8 | 1.8 | 1.7 | 1.6 | 1.6 | 1.5 | 1.4 | 1.3 |
| **1.66–1.70** | 0.9 | 1.1 | 1.3 | 1.4 | 1.6 | 1.7 | 1.8 | 1.9 | 1.9 | 1.8 | 1.8 | 1.7 | 1.6 | 1.5 | 1.4 | 1.4 |
| **1.71–1.75** | 0.9 | 1.1 | 1.3 | 1.5 | 1.7 | 1.8 | 1.9 | 1.9 | 1.9 | 1.9 | 1.8 | 1.7 | 1.6 | 1.5 | 1.5 | 1.4 |
| **1.76–1.80** | 0.9 | 1.1 | 1.3 | 1.5 | 1.7 | 1.8 | 1.9 | 2.0 | 2.0 | 1.9 | 1.9 | 1.8 | 1.7 | 1.6 | 1.5 | 1.4 |
| **1.81–1.85** | 0.9 | 1.1 | 1.4 | 1.6 | 1.8 | 1.9 | 2.0 | 2.0 | 2.0 | 2.0 | 1.9 | 1.8 | 1.7 | 1.6 | 1.5 | 1.5 |
| **1.86–1.90** | 1.0 | 1.2 | 1.4 | 1.6 | 1.8 | 1.9 | 2.0 | 2.0 | 2.0 | 2.0 | 2.0 | 1.9 | 1.8 | 1.7 | 1.6 | 1.5 |
| **1.91–1.95** | 1.0 | 1.2 | 1.4 | 1.7 | 1.9 | 2.0 | 2.0 | 2.0 | 2.0 | 2.0 | 2.0 | 1.9 | 1.8 | 1.7 | 1.6 | 1.6 |
| **1.96–2.00** | 1.0 | 1.2 | 1.5 | 1.7 | 1.9 | 2.0 | 2.0 | 2.0 | 2.0 | 2.0 | 2.0 | 2.0 | 1.9 | 1.8 | 1.7 | 1.6 |
| **2.01–2.05** | 1.0 | 1.3 | 1.5 | 1.7 | 1.9 | 2.0 | 2.0 | 2.0 | 2.0 | 2.0 | 2.0 | 2.0 | 1.9 | 1.8 | 1.7 | 1.6 |
| **2.06–2.10** | 1.1 | 1.3 | 1.6 | 1.8 | 2.0 | 2.0 | 2.0 | 2.0 | 2.0 | 2.0 | 2.0 | 2.0 | 2.0 | 1.9 | 1.8 | 1.7 |
| **2.11–2.15** | 1.1 | 1.3 | 1.6 | 1.8 | 2.0 | 2.0 | 2.0 | 2.0 | 2.0 | 2.0 | 2.0 | 2.0 | 2.0 | 1.9 | 1.8 | 1.7 |
| **2.16–2.20** | 1.1 | 1.4 | 1.6 | 1.9 | 2.0 | 2.0 | 2.0 | 2.0 | 2.0 | 2.0 | 2.0 | 2.0 | 2.0 | 1.9 | 1.8 | 1.8 |
| **2.21–2.25** | 1.1 | 1.4 | 1.7 | 1.9 | 2.0 | 2.0 | 2.0 | 2.0 | 2.0 | 2.0 | 2.0 | 2.0 | 2.0 | 2.0 | 1.9 | 1.8 |
| **2.26–2.30** | 1.2 | 1.4 | 1.7 | 2.0 | 2.0 | 2.0 | 2.0 | 2.0 | 2.0 | 2.0 | 2.0 | 2.0 | 2.0 | 2.0 | 1.9 | 1.8 |
| **2.31–2.35** | 1.2 | 1.5 | 1.7 | 2.0 | 2.0 | 2.0 | 2.0 | 2.0 | 2.0 | 2.0 | 2.0 | 2.0 | 2.0 | 2.0 | 2.0 | 1.9 |
| **2.36–2.40** | 1.2 | 1.5 | 1.8 | 2.0 | 2.0 | 2.0 | 2.0 | 2.0 | 2.0 | 2.0 | 2.0 | 2.0 | 2.0 | 2.0 | 2.0 | 1.9 |
| **2.41–2.45** | 1.2 | 1.5 | 1.8 | 2.0 | 2.0 | 2.0 | 2.0 | 2.0 | 2.0 | 2.0 | 2.0 | 2.0 | 2.0 | 2.0 | 2.0 | 1.9 |
| **2.46–2.50** | 1.3 | 1.5 | 1.8 | 2.0 | 2.0 | 2.0 | 2.0 | 2.0 | 2.0 | 2.0 | 2.0 | 2.0 | 2.0 | 2.0 | 2.0 | 2.0 |

**Table S1C. Activity calculator** **for patients with 16–20% liver-to-lung shunt ratio.** Activity values are given in GBq. BSA = body surface area.

| **BSA** | **Percentage tumour involvement (%)** | | | | | | | | | | | | | | | |
| --- | --- | --- | --- | --- | --- | --- | --- | --- | --- | --- | --- | --- | --- | --- | --- | --- |
|  | **0**–**5** | **6**–**10** | **11**–**15** | **16**–**20** | **21**–**25** | **26**–**30** | **31**–**35** | **36**–**40** | **41**–**45** | **46**–**50** | **51**–**55** | **56**–**60** | **61**–**65** | **66**–**70** | **71**–**75** | **76**–**80** |
| **1.30–1.35** | 0.7 | 0.8 | 1.0 | 1.2 | 1.3 | 1.4 | 1.5 | 1.5 | 1.5 | 1.5 | 1.4 | 1.3 | 1.3 | 1.2 | 1.1 | 1.1 |
| **1.36–1.40** | 0.7 | 0.9 | 1.0 | 1.2 | 1.3 | 1.4 | 1.5 | 1.5 | 1.5 | 1.5 | 1.5 | 1.4 | 1.3 | 1.2 | 1.2 | 1.1 |
| **1.41–1.45** | 0.7 | 0.9 | 1.1 | 1.2 | 1.4 | 1.5 | 1.5 | 1.5 | 1.5 | 1.5 | 1.5 | 1.4 | 1.4 | 1.3 | 1.2 | 1.2 |
| **1.46–1.50** | 0.8 | 0.9 | 1.1 | 1.3 | 1.4 | 1.5 | 1.5 | 1.5 | 1.5 | 1.5 | 1.5 | 1.5 | 1.4 | 1.3 | 1.3 | 1.2 |
| **1.51–1.55** | 0.8 | 1.0 | 1.1 | 1.3 | 1.5 | 1.5 | 1.5 | 1.5 | 1.5 | 1.5 | 1.5 | 1.5 | 1.5 | 1.4 | 1.3 | 1.2 |
| **1.56–1.60** | 0.8 | 1.0 | 1.2 | 1.4 | 1.5 | 1.5 | 1.5 | 1.5 | 1.5 | 1.5 | 1.5 | 1.5 | 1.5 | 1.4 | 1.3 | 1.3 |
| **1.61–1.65** | 0.8 | 1.0 | 1.2 | 1.4 | 1.5 | 1.5 | 1.5 | 1.5 | 1.5 | 1.5 | 1.5 | 1.5 | 1.5 | 1.5 | 1.4 | 1.3 |
| **1.66–1.70** | 0.9 | 1.1 | 1.3 | 1.4 | 1.5 | 1.5 | 1.5 | 1.5 | 1.5 | 1.5 | 1.5 | 1.5 | 1.5 | 1.5 | 1.4 | 1.4 |
| **1.71–1.75** | 0.9 | 1.1 | 1.3 | 1.5 | 1.5 | 1.5 | 1.5 | 1.5 | 1.5 | 1.5 | 1.5 | 1.5 | 1.5 | 1.5 | 1.5 | 1.4 |
| **1.76–1.80** | 0.9 | 1.1 | 1.3 | 1.5 | 1.5 | 1.5 | 1.5 | 1.5 | 1.5 | 1.5 | 1.5 | 1.5 | 1.5 | 1.5 | 1.5 | 1.4 |
| **1.81–1.85** | 0.9 | 1.1 | 1.4 | 1.5 | 1.5 | 1.5 | 1.5 | 1.5 | 1.5 | 1.5 | 1.5 | 1.5 | 1.5 | 1.5 | 1.5 | 1.5 |
| **1.86–1.90** | 1.0 | 1.2 | 1.4 | 1.5 | 1.5 | 1.5 | 1.5 | 1.5 | 1.5 | 1.5 | 1.5 | 1.5 | 1.5 | 1.5 | 1.5 | 1.5 |
| **1.91–1.95** | 1.0 | 1.2 | 1.4 | 1.5 | 1.5 | 1.5 | 1.5 | 1.5 | 1.5 | 1.5 | 1.5 | 1.5 | 1.5 | 1.5 | 1.5 | 1.5 |
| **1.96–2.00** | 1.0 | 1.2 | 1.5 | 1.5 | 1.5 | 1.5 | 1.5 | 1.5 | 1.5 | 1.5 | 1.5 | 1.5 | 1.5 | 1.5 | 1.5 | 1.5 |
| **2.01–2.05** | 1.0 | 1.3 | 1.5 | 1.5 | 1.5 | 1.5 | 1.5 | 1.5 | 1.5 | 1.5 | 1.5 | 1.5 | 1.5 | 1.5 | 1.5 | 1.5 |
| **2.06–2.10** | 1.1 | 1.3 | 1.5 | 1.5 | 1.5 | 1.5 | 1.5 | 1.5 | 1.5 | 1.5 | 1.5 | 1.5 | 1.5 | 1.5 | 1.5 | 1.5 |
| **2.11–2.15** | 1.1 | 1.3 | 1.5 | 1.5 | 1.5 | 1.5 | 1.5 | 1.5 | 1.5 | 1.5 | 1.5 | 1.5 | 1.5 | 1.5 | 1.5 | 1.5 |
| **2.16–2.20** | 1.1 | 1.4 | 1.5 | 1.5 | 1.5 | 1.5 | 1.5 | 1.5 | 1.5 | 1.5 | 1.5 | 1.5 | 1.5 | 1.5 | 1.5 | 1.5 |
| **2.21–2.25** | 1.1 | 1.4 | 1.5 | 1.5 | 1.5 | 1.5 | 1.5 | 1.5 | 1.5 | 1.5 | 1.5 | 1.5 | 1.5 | 1.5 | 1.5 | 1.5 |
| **2.26–2.30** | 1.2 | 1.4 | 1.5 | 1.5 | 1.5 | 1.5 | 1.5 | 1.5 | 1.5 | 1.5 | 1.5 | 1.5 | 1.5 | 1.5 | 1.5 | 1.5 |
| **2.31–2.35** | 1.2 | 1.5 | 1.5 | 1.5 | 1.5 | 1.5 | 1.5 | 1.5 | 1.5 | 1.5 | 1.5 | 1.5 | 1.5 | 1.5 | 1.5 | 1.5 |
| **2.36–2.40** | 1.2 | 1.5 | 1.5 | 1.5 | 1.5 | 1.5 | 1.5 | 1.5 | 1.5 | 1.5 | 1.5 | 1.5 | 1.5 | 1.5 | 1.5 | 1.5 |
| **2.41–2.45** | 1.2 | 1.5 | 1.5 | 1.5 | 1.5 | 1.5 | 1.5 | 1.5 | 1.5 | 1.5 | 1.5 | 1.5 | 1.5 | 1.5 | 1.5 | 1.5 |
| **2.46–2.50** | 1.3 | 1.5 | 1.5 | 1.5 | 1.5 | 1.5 | 1.5 | 1.5 | 1.5 | 1.5 | 1.5 | 1.5 | 1.5 | 1.5 | 1.5 | 1.5 |
